# Frequency modulation of entorhinal cortex neuronal activity drives distinct frequency-dependent states of brain-wide dynamics

**DOI:** 10.1016/j.celrep.2021.109954

**Published:** 2021-11-02

**Authors:** Piergiorgio Salvan, Alberto Lazari, Diego Vidaurre, Francesca Mandino, Heidi Johansen-Berg, Joanes Grandjean

**Affiliations:** 1 Wellcome Centre for Integrative Neuroimaging, FMRIB, Nuffield Department of Clinical Neurosciences, University of Oxford, Oxford OX3 9DU, UK; 2 Wellcome Centre for Integrative Neuroimaging, OHBA, Department of Psychiatry, University of Oxford, Oxford OX3 7JX, UK; 3 Department of Clinical Medicine, Center for Functionally Integrative Neuroscience, Aarhus University, Aarhus 8000, Denmark; 4 Department of Radiology and Biomedical Imaging, Yale University School of Medicine, New Haven, CT 06520, USA; 5 Department of Medical Imaging and Donders Institute for Brain, Cognition, and Behaviour, Donders Institute, Radboud University Medical Centre, PO Box 9101, 6500HB Nijmegen, the Netherlands

**Keywords:** requency modulation, dynamic brain networks, optogenetics-fMRI, hidden Markov modeling

## Abstract

Human neuroimaging studies have shown that, during cognitive processing, the brain undergoes dynamic transitions between multiple, frequency-tuned states of activity. Although different states may emerge from distinct sources of neural activity, it remains unclear whether single-area neuronal spiking can also drive multiple dynamic states. In mice, we ask whether frequency modulation of the entorhinal cortex activity causes dynamic states to emerge and whether these states respond to distinct stimulation frequencies. Using hidden Markov modeling, we perform unsupervised detection of transient states in mouse brain-wide fMRI fluctuations induced via optogenetic frequency modulation of excitatory neurons. We unveil the existence of multiple, frequency-dependent dynamic states, invisible through standard static fMRI analyses. These states are linked to different anatomical circuits and disrupted in a frequency-dependent fashion in a transgenic model of cognitive disease directly related to entorhinal cortex dysfunction. These findings provide cross-scale insight into basic neuronal mechanisms that may underpin flexibility in brain-wide dynamics.

## Highlights

• Frequency modulation of the entorhinal cortex (EC) via optogenetics-fMRI
• Hidden Markov modeling reveals the emergence of multiple dynamic brain states
• Distinct dynamic states preferentially respond to different theta frequencies
• A transgenic model of EC network dysfunction disrupts frequency-dependent responses

## Graphical abstract

**Figure:**
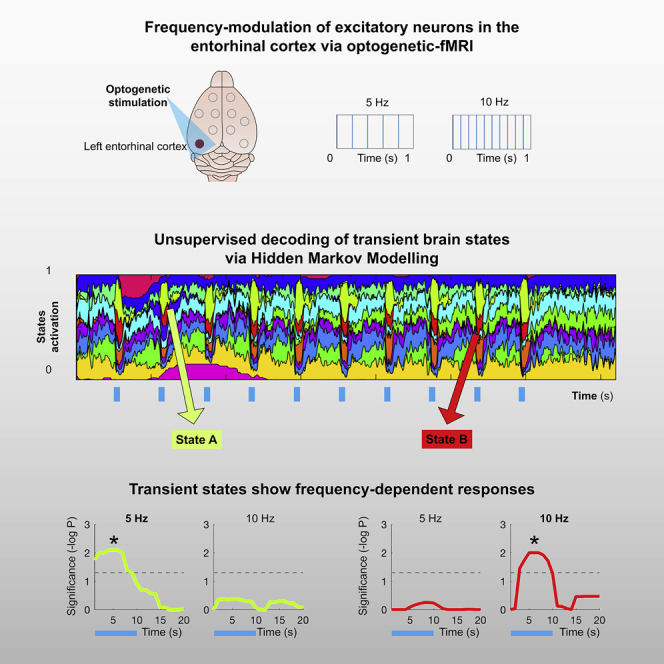

## Teaser

Frequency modulation of the entorhinal cortex (EC) via optogenetics-fMRI allows the establishment of a causal link between cell-specific neuronal activity and brain-wide dynamics. Salvan et al. demonstrate how multiple, frequency-dependent dynamic states can emerge from theta modulation of excitatory neurons in the EC.

## Introduction

There is increasing evidence that in the human brain, changes in online cognitive processing are coupled with dynamic states of brain function (van der Meer et al., 2020; Quinn et al., 2018). Although anatomically they resemble canonical brain networks (Smith et al., 2019), these transient, repeating states are characterized by fast, frequency-specific changes in brain activity and connectivity that take place over a few hundred milliseconds (Baker et al., 2014; Vidaurre et al., 2018). These dynamic states are also present at rest (Vidaurre et al., 2018) and during replay bursts (Higgins et al., 2021), and are thus likely to perform a variety of computational and behavioral roles. Despite their importance to brain function and behavior, the investigation into the mechanisms supporting dynamic states has been restricted to human neuroimaging, thus limiting our understanding of their underlying cellular basis. Understanding what cellular mechanisms may drive the emergence of dynamic brain states is key to understanding the cellular basis of flexible cognition.

Although these dynamic states unfold over time across the whole brain, it is unclear whether they emerge from global activity patterns or whether activity in an individual area is sufficient to drive their emergence. Furthermore, although distinct brain states may originate from multiple sources of neural activity, whether multiple dynamic states can also be induced by a single-area neuronal spiking remains unknown. Human studies (Higgins et al., 2021; Vidaurre et al., 2018) suggest that frequency modulation of neuronal activity may be one of the key mechanisms underlying the transient organization of these brain states. However, this hypothesis can only be confirmed through targeted causal intervention methods, such as those available in rodent models. However, previous photostimulation studies in rodents have commonly neglected transient brain-wide events. These studies have therefore overwhelmingly shown that frequency modulation of neuronal activity results in monotonic, static changes in brain function (Chan et al., 2017; Leong et al., 2016; Liu et al., 2015), but they have not explored whether it elicits multiple, transient, and frequency-dependent states. This leaves the question open as to whether neuronal frequency modulation is a causal driver of dynamic states.

Given its brain-wide projections and the preferential modulation in the narrow but heterogeneous theta band (4–12 Hz) (Chrobak et al., 2000; Mizuseki et al., 2009), the entorhinal-hippocampal circuit represents an ideal system to test whether frequency modulation in an individual brain area can drive distinct dynamic states. In this work, we ask whether frequency modulation of the entorhinal cortex (EC) activity in mice causes dynamic states to emerge, and whether these dynamic states respond specifically to distinct stimulation frequencies. Functionally, the rodent hippocampus displays multiple forms of theta oscillations at various frequencies (Goutagny et al., 2009; Mikulovic et al., 2018): high theta (in rodents defined as type 1; 7–12 Hz), and low theta (type 2; 4–9 Hz). This multiplicity in theta oscillations is also present in humans (Goyal et al., 2020), and suggests that high and low theta activity may be able to elicit distinct, frequency-specific dynamic brain states.

Here, we hypothesize that theta frequency modulation of channelrhodopsin-2 (ChR2)-transfected excitatory neurons in the EC (Boyden et al., 2005) can elicit distinct dynamic states of downstream projection activity, and that these dynamic states respond in a frequency-dependent manner. To test this, we used blood oxygen level-dependent (BOLD) fMRI fluctuations to record mouse brain-wide activity during optogenetic modulation of the EC at 5, 10, and 20 Hz. We then applied hidden Markov models (HMMs) to characterize transient, recurring, dynamic patterns of activity, referred to as states, in a completely data-driven fashion. By combining HMM-fMRI with concurrent optogenetic modulation of the EC, we demonstrate that EC neuronal spiking is sufficient to elicit at least two temporally overlapping but distinct, frequency-dependent dynamic states. These states were preferentially engaged (were significantly more active) at different theta rhythms (either 5 or 10 Hz, but not 20 Hz), providing causal evidence that frequency modulation of a single brain area is sufficient to elicit multiple states of dynamic brain function. Finally, we test frequency-dependent network engagement in a transgenic model of EC dysfunction. The 3xTgAD mouse model for Alzheimer’s disease-like pathology (Oddo et al., 2003) shows phospho-tau accumulation in post-synaptic targets of the EC, namely hippocampus and basolateral amygdala, as well as electrophysiological signatures indicating increased EC neuronal excitability (Mandino et al., 2020). This pathological manifestation leads to clear behavioral deficits, for example, in episodic-like memory, fear processing (Davis et al., 2014; España et al., 2010), and retention/retrieval deficits (Stover et al., 2015). The model thus allows us to provide additional evidence of the biological relevance of the frequency-dependent brain states characterized here, by showing dynamic brain state disruption in a transgenic model of cognitive disease directly related to EC network dysfunction.

## Results

### Single-area evoked neuronal spiking is sufficient to cause distinct, transient states of brain activity and connectivity

From a modeling perspective, it is possible to use the sequential nature of BOLD-fMRI data to characterize transient dynamic states in brain hemodynamic activity. HMMs are well suited because they provide a time-point-by-time-point description of brain activity, facilitating the characterization of how such responses may evolve across time (Vidaurre et al., 2017) or in response to perturbations such as stimulation.

**Figure 1:**
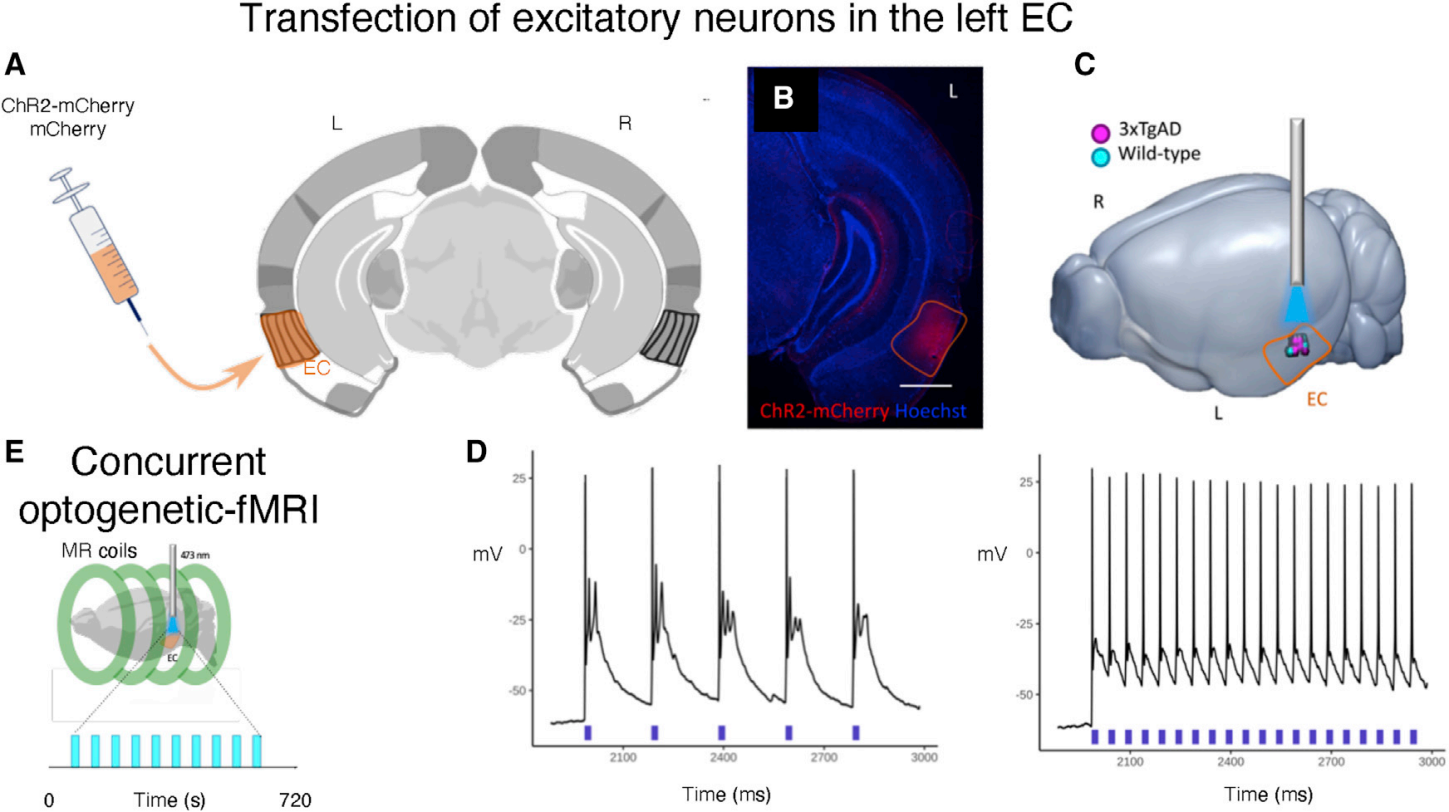
Optogenetic stimulation of the excitatory neurons in the EC and concurrent whole-brain fMRI (A) Schematic illustrating CaMKII-ChR2-mCherry and CaMKII-mCherry transfection in the left lateral entorhinal cortex (EC). Coordinates relative to bregma and midline: −2.8 mm from bregma and +4.2 mm from the midline; depth of the injection *in loco:* −2.8/−2.6 mm. (B) Histological validation confirms ChR2-mCherry expression (red) in the EC, with counterstain with DAPI (blue) for cell nucleus. (C) Three-dimensional (3D) rendering of the optrode stimulation targets clustered within the EC in both wild-type (WT) control mice (cyan dots) and 3xTgAD (pink dots). (D) Lightly anesthetized mice under mechanical ventilation underwent high-field fMRI acquisition runs, concurrently with 10 optogenetic photostimulation blocks of 10 s interleaved with 50 s inter-stimuli intervals per run. Photostimulation was conducted in the left lateral entorhinal cortex with frequencies of 5, 10, and 20 Hz, pseudo-randomized between runs. (E) Photostimulation of EC neurons elicited a faithful response to both (left) 5 Hz and (right) 20 Hz stimulation pulses, here shown in WT mice, as confirmed in *ex vivo* slice preparation.

Here, we combined HMM with optogenetics-evoked fMRI (ofMRI) (Leong et al., 2019) in lightly anesthetized mice with ChR2 transfected into excitatory neurons of the EC (Boyden et al., 2005). Photostimulation was conducted with frequencies of 5, 10, and 20 Hz, pseudo-randomized between runs (Figure 1). Transfected EC neurons faithfully responded with frequency-locked action potentials to both extremes of the frequency-range tested (Figure 1E).

**Figure 2:**
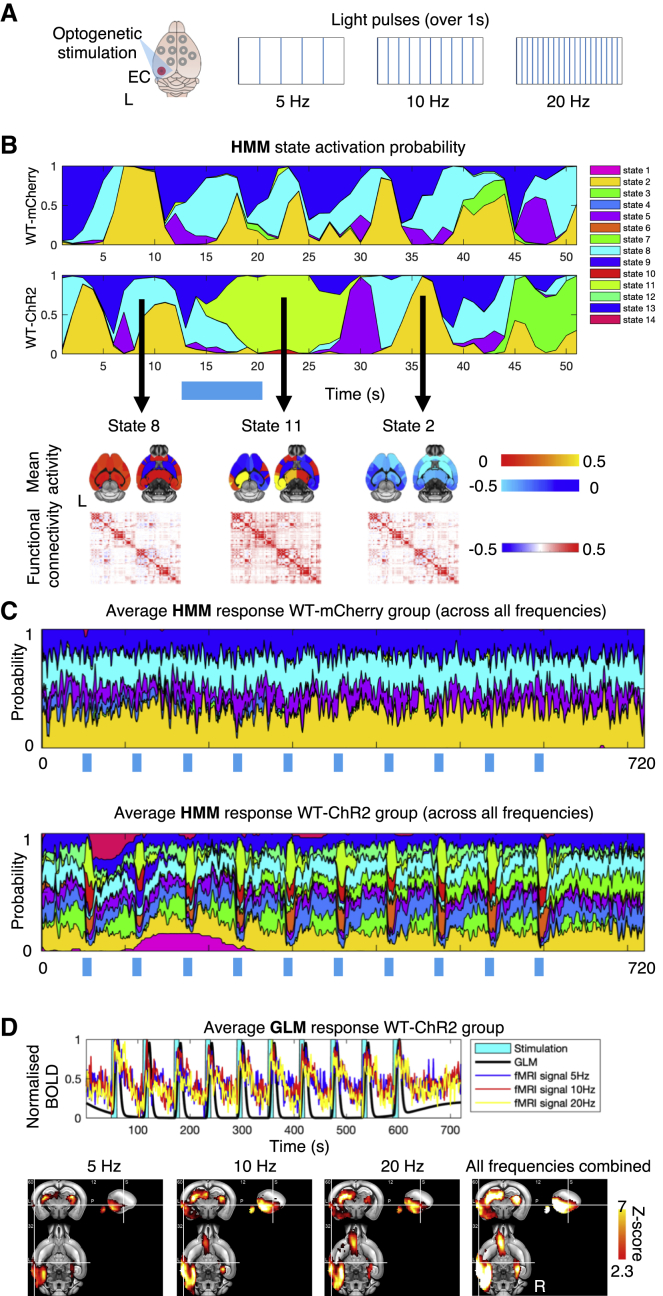
Hidden Markov modeling detects dynamic states of brain activity and connectivity during optogenetic stimulation of excitatory neurons in the EC Whole-brain fMRI BOLD fluctuations in response to optogenetic stimulation were modeled through hidden Markov models (HMMs), characterizing a discrete number of dynamic states. Temporally, they are represented by a specific state time course that for each subject indicates when each state is active (activation probability). Spatially, each state is characterized by a mean activation map (BOLD amplitude) and by a functional connectivity matrix (BOLD covariance). (A) Light pulses at 3 different frequencies were used for optogenetic stimulation. Here, light traces are displayed for a period of 1 s. (B) A 50-s section of the state time course for 1 example subject. Top: states activation probability for 1 WT-mCherry mouse and 1 WT-ChR2 mouse. Bottom: state-specific mean activation map (negative values indicate activity below average brain amplitude) and functional connectivity matrix. A 14-state HMM was used to study dynamic states in response to EC stimulation at 5, 10, and 20 Hz in 3 groups of mice: N = 31 mice (N_WT-mCherry_ = 9, N_WT-ChR2_ = 10, N_3xTgAD-ChR2_ = 12), n = 153 runs (see Figure S1 for details on HMM fit). (C) Average HMM response for WT-mCherry group (top) and WT-ChR2 group (bottom) aligned with optogenetic stimulation blocks. Optogenetic stimulation blocks are shown as a blue bar underneath HMM states time courses. (D) GLM modeling of BOLD fMRI response to optogenetic stimulation. Top: results from analyzing only the WT-ChR2 group. Bottom: showing 2D brain maps depicting activation as z statistic level, thresholded at family-wise error-corrected (FWE-corr) p < 0.05. Brain activation maps for stimulating EC at 5, 10, and 20 Hz, and for all frequencies combined, respectively.

We tested the hypothesis that localized excitatory neuronal activity modulated at distinct stimulation frequencies (5, 10, and 20 Hz) (Figure 2A) causes multiple, transient states of large-scale hemodynamic activity. By applying HMMs on temporally concatenated time-series data from subjects across experimental groups (N total = 31 mice: N_WT-mCherry_ = 9, N_WT-ChR2_ = 10, N_3xTgAD-ChR2_ = 12; n total = 153 ofMRI runs), we sought to describe brain hemodynamics as a collection of 14 dynamic states (which yielded greater model similarity and lower free energy compared to other numbers of states; Figure S1A). Each state captures a unique pattern of both amplitude and functional connectivity across brain regions (Figure 2B and S1D). Although the HMM states parameters were inferred at the group level, each animal has its own characteristic states’ time courses representing the subject-specific probability of each HMM state being active at each instant (here, an fMRI volume). This 14-state HMM was then used to compare differences in dynamic states responses to optogenetic stimulation in the ChR2 and control groups (N_WT-ChR2_ = 10 mice; n = 54 runs; each animal underwent 6 runs acquired in 2 sessions; mCherry; N_WT-mCherry_ = 9 mice, n = 27 runs; each animal underwent 3 runs acquired in 1 session; all stimulation frequencies combined). Although HMM is an unsupervised method with no a priori knowledge of the stimulation paradigm, it was able to characterize changes in fast brain dynamics in response to the optogenetic stimulation in ChR2 animals (Figure 2C; replicated with different states numbers as shown in Figures S2A and S2B). As a comparison, a standard approach relying on mass-univariate general linear model (GLM) testing yielded a single pattern of static spatial BOLD activity (and no knowledge of changes in static or dynamic functional connectivity) in response to the optogenetic stimulation (Figure 2D).

**Figure 3:**
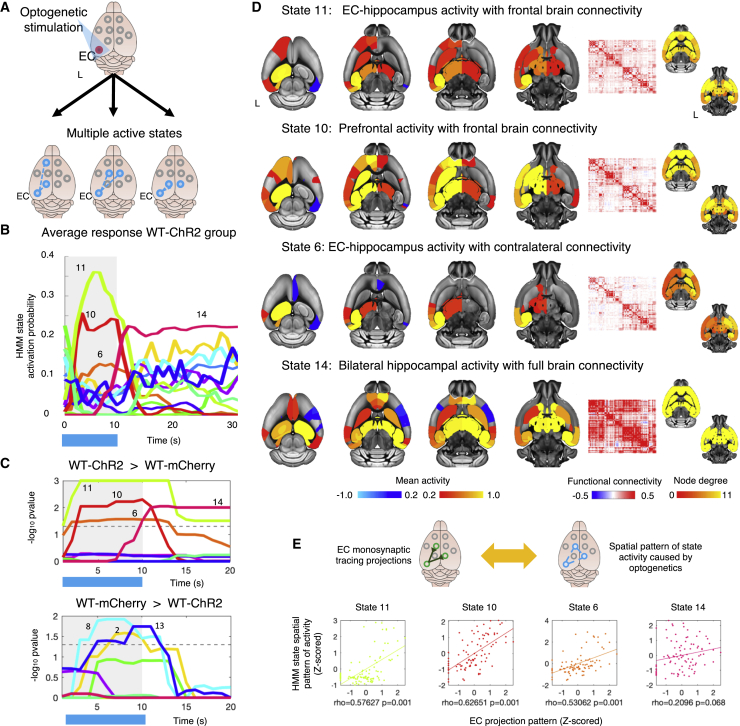
Optogenetic stimulation of the EC causes distinct dynamic states (A) Hidden Markov modeling of concurrent optogenetic fMRI (ofMRI) was used to test whether inducing frequency modulation of neuronal activity in a single brain area is sufficient to cause multiple states of brain activity. (B) Average HMM activation probability plotted via activation lines and time-locked to optogenetic stimulation (shown by blue bar underneath) for WT-ChR2 group and across runs and stimulation blocks. (C) −Log_10_ FWE-corr p values (across time and states) of group difference in HMM states activation probability time locked to optogenetic stimulation (shown by blue bar and gray background). The threshold for statistical significance is indicated by the dotted line. Sample: WT-mCherry control group (N_WT-mCherry_ = 9 mice, n = 27 runs; each animal underwent 3 runs acquired in 1 session, all stimulation frequencies combined); and WT-ChR2 group (N_WT-ChR2_ = 10 mice; n = 54 runs; each animal underwent 6 runs acquired in 2 sessions, all stimulation frequencies combined). (D) Spatial maps of the 4 HMM states found to be significantly more active in the WT-ChR2 group compared to WT-mCherry control. Each state was modeled with both an activation map (left) and a functional connectivity matrix (right). (E) We then tested whether the spatial patterns of state activity caused by optogenetics could be explained by EC monosynaptic tracing projections. Showing scatterplots of association between EC monosynaptic projection pattern and spatial pattern of HMM states mean activity (p values are FWE-corr).

We then looked at the evoked dynamic state response to time-locked optogenetic stimulation (Figure 3). Across blocks, for each time point, we tested whether the probability of each HMM state being active was greater in the group expressing ChR2 compared to the mCherry controls. During stimulation, we found that 3 states (states 11, 10, and 6) were significantly more active in the ChR2 group, compared to controls (while correcting across time and states; Figure 3C, top). Notably, the spatial pattern of activity of these 3 states was significantly associated with the pattern of the monosynaptic projection of EC, suggesting that these amplitude changes are direct results of inducing activation in excitatory neurons in EC (Figures 3E and S3A). All 3 states significantly respond to optogenetic stimulation showing overlapping temporal responses. With a delayed onset, rise time of ∼5–7 s, and slower decay, the HMM responses evoked by ofMRI matched the dynamics of conventional GLM-based analysis (Figure 2D). State 11 (Figure 3D) shows EC-hippocampus activity with bilateral frontal and hippocampal brain connectivity; state 10 shows prefrontal activity with frontal and hippocampal brain connectivity; and state 6 shows EC-hippocampus activity with contralateral connectivity. These findings provide forward, causal evidence that inducing the activation of excitatory neurons with multiple frequencies is sufficient to induce distinct dynamic states.

We also found that 3 states (states 2, 8, and 13) were significantly more active in the control group compared to the ChR2 group during optogenetic stimulation (Figure 3C, bottom). However, as clearly shown in Figures 2B and 2C (group average of HMM activation probability for the mCherry group), in the control group, these states are not time locked to stimulation. Rather, these group differences are the results of states 2, 8, and 13 de-activating in the ChR2 animals during stimulation. In the control group, states 2, 8, and 13 are active throughout the whole ofMRI run, and therefore represent states of resting brain activity. Of interest, we were also able to show that in ChR2-transfected animals, 1 state (state 14) showed significantly greater activation after the end of the first stimulation; this state was significantly present in ChR2-transfected animals but not in controls. State 14 showed bilateral hippocampal activity with full brain connectivity (Figure 3D). This result suggests that after the activation of excitatory neurons via optogenetic stimulation, the brain does not necessarily return to a baseline state. Previous work, studying the optogenetic activation of the motor cortex, reported robust, delayed hemodynamic (and electrical) responses in the thalamus, an area with known axonal projections from the motor cortex (Lee et al., 2010). Downstream, delayed non-local activity is likely to depend upon a cascade effect that involves neuronal as well as non-neuronal elements, giving rise to more complex contributions to the vascular response (Logothetis, 2010). It is possible that we observed in the present work how induced neuronal spiking in genetically targeted cells is sufficient to trigger a series of cascade effects, resulting in entrainment into a non-baseline state, represented by state 14.

As well as highlighting short-scale, time-locked responses to inducing neuronal activity, combining HMM with ofMRI allowed us to characterize long-scale variations in responses (state 6): how dynamic state responses to individual stimulation blocks varied over the course of the entire session (Figures S3B and S3C). Therefore, it is possible that local feedforward and feedback mechanisms may modulate the unfolding of brain dynamics over time, creating long-scale changes across blocks. At a microscopic level, these mechanisms may be mediated by strong axon collaterals, interacting in a recurrent excitatory feedback loop (Douglas and Martin, 2007), which are controlled by a variety of GABAergic interneurons (Markram et al., 2004) and are known to give rise to a recurrent ensemble of excitation and inhibition.

### Distinct dynamic states caused by EC spiking show frequency-dependent responses within the theta frequency band

Neuronal oscillations based on theta modulation represent the basis of EC-hippocampal circuit communication and are thought to support memory encoding and recall (Chrobak et al., 2000; Mizuseki et al., 2009). Here, we tested the hypothesis that inducing activity with distinct neuronal spiking rates (within the theta rhythms—thus 5 and 10 Hz, but not 20 Hz) on top of a single-brain area basal activity would cause distinct dynamic brain states to emerge and to preferentially respond at different theta frequencies (either 5 or 10 Hz).

**Figure 4:**
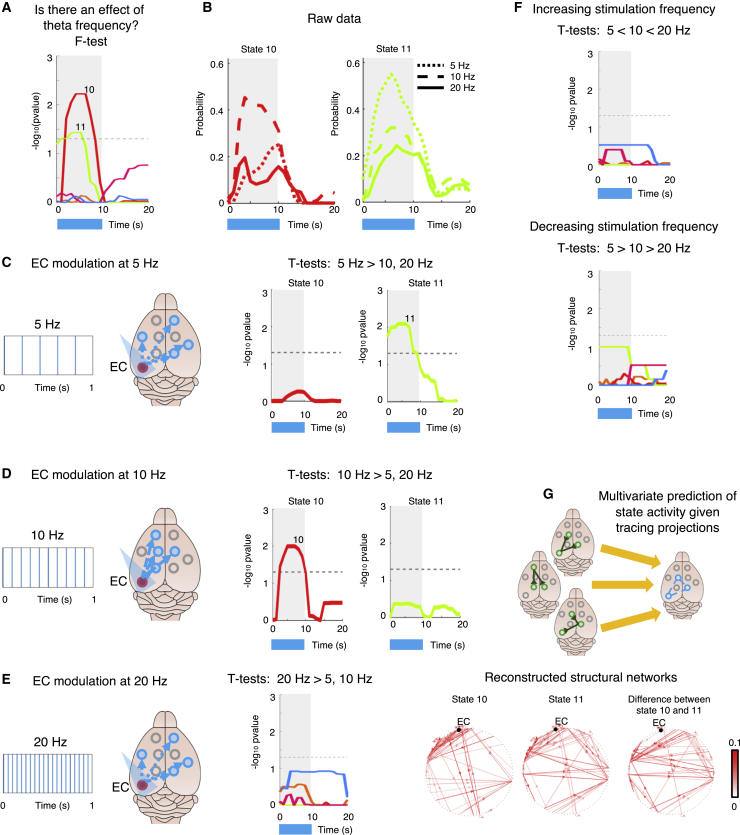
Distinct dynamic states show frequency-dependent responses within the theta frequency band We tested the hypothesis that distinct dynamic states show frequency-dependent responses (exemplified in diagrams in C and E). Results from analyzing only the WT-ChR2 group (N_WT-ChR2_ = 10 mice; n = 54 runs; each animal underwent 2 sessions of 3 runs, one run per stimulation frequency (5, 10, 20Hz)). Stimulation frequencies were explicitly modeled in the statistical tests. A repeated-measure ANOVA was used in order to test the effect of varying stimulation frequency on HMM states activation probability. (A) −Log_10_ FWE-corr p values for an overall effect of frequency on HMM states activation probability (F-test results). (B) Raw data: HMM activation probability for states 10 (displayed in red) and 11 (displayed in green) during stimulation at 5, 10, and 20 Hz. (C and D) Results from repeated-measures ANOVA post hoc t tests (as −log_10_ FWE-corr p values) of the 2 contrasts 5 Hz > 10, 20 Hz (C) and 10 Hz > 5, 20 Hz (D), for both state 10 (in red) and state 11 (in green). (E) Control test: results from t test (as −log_10_ FWE-corr p values) of the contrasts 20 Hz > 5, 10 Hz. (F) Control test: results from t tests (as −log_10_ FWE-corr p values) of the 2 contrasts 5 Hz < 10 Hz < 20 Hz (top) and 5 Hz > 10 Hz > 20 Hz (bottom). (G) To highlight the structural networks underlying states showing frequency-specific responses (C and D), we tested whether states 10 and 11 spatial activity could be predicted using multivariate tracing projections. Top: diagram. Bottom: predicted structural networks underlying the activity pattern of states 10 and 11 and the difference between the 2 (see Figure S4 for details). The color bar indicates the strength of the anatomical connections. For (A) and (C)–(F), the dotted line indicates the statistical threshold.

In the wild-type (WT)-ChR2 group (N_WT-ChR2_ = 10 mice; n = 54 runs; each animal underwent 2 sessions of 3 runs, 1 run per stimulation frequency [5, 10, and 20 Hz]), we focused on the HMM states, which showed time-locked increases in response to stimulation (states 6, 10, 11, and 14), and assessed their response to stimulation at 5 Hz (compared to 10 and 20 Hz) and at 10 Hz (compared to 5 and 20 Hz). Using a repeated-measures ANOVA (see Method details), we tested the effect of frequency on HMM state probability onset. We found a significant overall (F-test) effect of inducing neuronal firing with theta rhythms on states 10 and 11 (Figures 4A and 4B). Crucially, the post hoc analyses (t tests) showed that while state 10 responded significantly more at 10 Hz stimulation (compared to 5 and 20 Hz), state 11 responded more at 5-Hz stimulation (compared to 10 and 20 Hz) (Figures 4C and 4D, replicated for different HMM states number in Figures S2C and S2D). As a control test, we tested whether these frequency-dependent effects were the result of a greater EC responsivity as a function of either increasing (5 Hz < 10 Hz < 20 Hz) or decreasing (5 Hz > 10 Hz > 20 Hz) stimulation frequency. Using a repeated-measures ANOVA, we tested for a monotonic increase or decrease in HMM states’ response: no significant result was found (Figure 4F). As a further control test, we tested for a specific response to 20-Hz stimulation (compared to 5 and 10 Hz); no significant result was found (Figure 4E). These results show that even in the narrow range of theta rhythms, there are at least 2 distinct transient states of brain activity that are preferentially engaged (significantly more active) at different theta frequencies (either 5 or 10 Hz). These findings causally link theta frequency modulation in excitatory neurons in the EC with distinct spatiotemporal patterns of brain-wide dynamics.

If distinct dynamic brain states preferentially respond to different modulation frequencies arising from a single source of neuronal activity, then a possibility could be that they rely on partially different anatomical circuits. To test this hypothesis, we performed structural-to-functional mapping (Figure 4G), predicting states’ activity pattern using the connectivity matrix of directed tracing projections publicly available from the Allen Brain Institute (Oh et al., 2014). Using leave-one-out cross-validated Ridge regression, we calculated out-of-sample explained variance for the activity pattern of both states 10 and 11 (Figures S4A–S4C: state 10: predicted rho = 0.82 (R^2^ = 0.67), deviance = 29.66, p = 0.001; Figures S4D–S4F: state 11: predicted rho = 0.77 (R^2^ = 0.60), deviance = 35.72, p = 0.001; significance assessed with 1,000 permutations). We then applied this approach to characterize the anatomical connections that better predicted the difference in activation patterns between states 10 and 11 (Figures S4G–S4I: difference between state 10 and 11: predicted rho = 0.68 (R^2^ = 0.47), deviance = 47.59, p = 0.001). We found that the main features indicated differences in directed anatomical connectivity mainly to the left caudoputamen and the left nucleus accumbens, suggesting that left anatomical connections targeting subcortical structures may partially underlie the functional differences observed between states 10 and 11. These results show the 2 directed structural circuits that may underlie activity in frequency-dependent HMM states and the main anatomical connections that characterize the difference in activation between these 2 states, suggesting that partially different anatomical circuits may provide an anatomical basis for distinct transient states of brain activity elicited by the same, single source of activity via frequency modulation.

### Frequency-dependent responses of brain states are disrupted in a transgenic model of EC dysfunction

**Figure 5:**
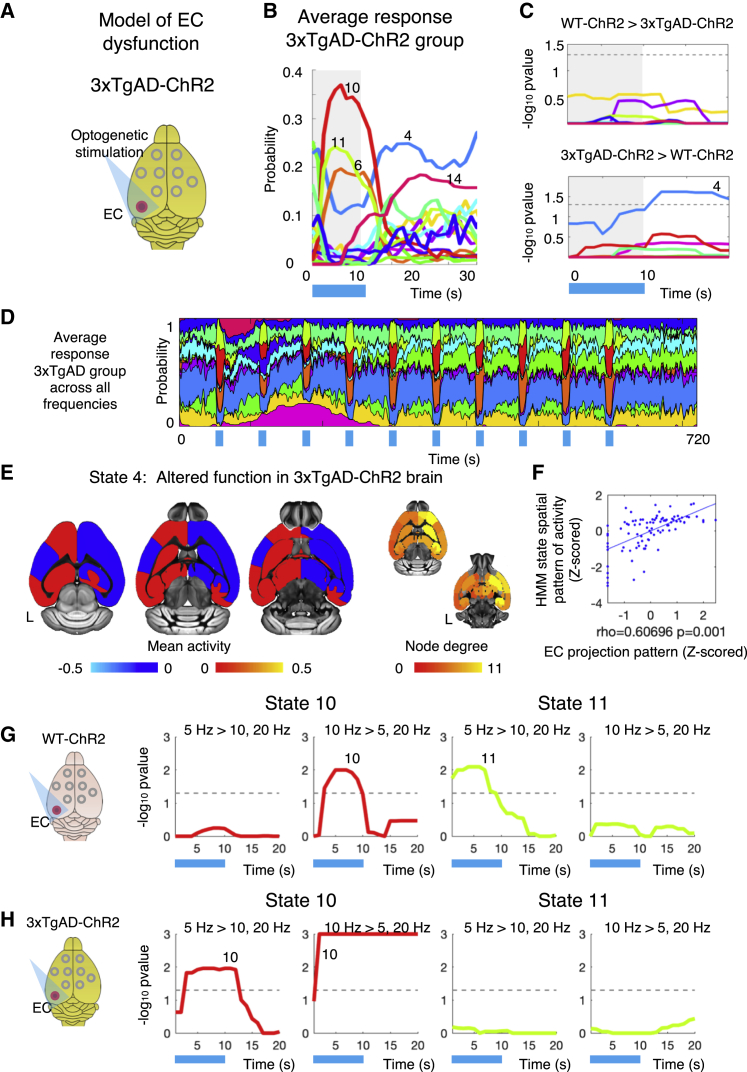
Frequency-specific responses of EC downstream activity are disrupted in a transgenic model of EC dysfunction (A) Diagram of MRI in 3xTgAD mice. (B) Average HMM activation probability plotted via activation lines and time locked to optogenetic stimulation (shown by blue bar underneath) for 3xTgAD-ChR2 group and across runs and stimulation blocks. (C) −Log_10_ FWE-corr p values (across time and states) of group difference between 3xTgAD-ChR2 and WT-ChR2 in HMM states activation probability. Dotted line indicates the statistical threshold. Top: WT-ChR2 > 3xTgAD-ChR2. Bottom: 3xTgAD-ChR2 > WT-ChR2. Statistical analyses were carried out on the WT-ChR2 group and the 3xTgAD-ChR2 group (N_WT-ChR2_ = 10 mice; n = 54 run; N_3xTgAD-ChR2_ = 12 mice; n = 72 runs; for both groups, each animal underwent 2 sessions of 3 runs, 1 run per stimulation frequency [5, 10, and 20 Hz]). (C) States 10, 11, and 6, known to be caused by optogenetics, are also present in this 3xTgAD model of EC disruption. (D) Average response for the 3xTgAD-ChR2 group aligned with optogenetic stimulation blocks. (E) Aberrant activity in 3xTgAD mice. Left: 2D brain map of aberrant activity. Right: 3D map of aberrant node degree. (F) Scatterplots of association between EC monosynaptic projection pattern and HMM state 4 spatial pattern of mean activity. (G and H) Results for single-group repeated-measures ANOVA post hoc t tests (as −log_10_ FWE-corr p values), respectively, for WT-ChR2 group (G) and for 3xTgAD-ChR2 group (H), testing for the effect of theta frequencies (5 Hz > 10, 20 Hz; and 10 Hz > 5, 20 Hz; showing in red the results for state 10 and in green the results for state 11). Together, these 2 panels show a statistically significant disruption of frequency-dependent state responses in the transgenic model of EC dysfunction (3xTgAD-ChR2 group), despite the existence of intact HMM states caused by optogenetic stimulation.

We then asked whether these frequency-dependent state responses to EC frequency modulation are impaired in a mouse model with known cellular alterations in the EC projection network (Figures 5A and 5B). We used a triple-transgenic mouse model for Alzheimer’s disease (3xTgAD) (Oddo et al., 2003) in which the presence of phospho-tau (a precursor for the hallmark tauopathy of neurofibrillary tangles) early in development (Mastrangelo and Bowers, 2008; Yeh et al., 2011) leads to EC damage. Using ofMRI, we tested whether HMM dynamic states are affected in the 3xTgAD mice compared to WT mice (N_WT-ChR2_ = 10 mice; n = 54 runs; each animal underwent 6 runs acquired in 2 sessions; 3xTgAD-ChR2, N_3xTgAD-ChR2_ = 12 mice; n = 72 runs, each animal underwent 6 runs acquired in 2 sessions; all stimulation frequencies combined). While many aspects of optogenetic stimulation-induced activity were intact (Figures 5C and 5D), we found an aberrant state of baseline activity (state 4) that showed a significant reduction in activation probability during stimulation in 3xTgAD mice (Figure 5E). The spatial pattern of state 4 activity was positively associated with EC monosynaptic projections (Figure S5F), suggesting that this baseline state of aberrant hyperactivity may reflect the increased electrophysiological facilitation caused by early tauopathy (Oddo et al., 2003). These results show that in this model of EC network dysfunction, despite the presence of a baseline state of aberrant activity that is downregulated during stimulation, the multiplicity of states caused by optogenetics and observed in the healthy WT group is not altered.

Then, building on the fact that distinct brain states preferentially respond to different EC stimulation theta frequencies in WT mice (see Figure 4), we tested whether these frequency-specific responses were different between 3xTgAD and WT mice (N_WT-ChR2_ = 10 mice; n = 54 runs; N_3xTgAD-ChR2_ = 12 mice; n = 72 runs; for both groups, each animal underwent 2 sessions of 3 runs, 1 run per stimulation frequency [5, 10, and 20 Hz]). Using a mixed-effect ANOVA (see Method details), we tested a group by theta frequencies interaction on HMM state probability onset. We found a significant overall interaction effect (F-test) of group by neuronal firing frequency in states 10 and 11 (Figure S5A–S5C). The post hoc interaction analyses (t tests) showed that while state 11 response at 5-Hz stimulation (compared to 10 and 20 Hz) was significantly greater in WT compared to 3xTgAD mice, the state 10 response was significantly greater in 3xTgAD compared to WT mice at both 5 and 10 Hz stimulation (compared to 20 Hz) (Figure S5D). These results show that while ofMRI states elicited in WT mice exist in 3xTgAD mice and do not differ (Figure 5C), brain state frequency-dependent responses to varying stimulation frequency differed significantly between groups. Specifically, while in WT mice different dynamic brain networks were shown to preferentially respond to varying stimulation frequencies (state 10 to 10 Hz and state 11 to 5 Hz), in 3xTgAD mice, state 10 showed a preferential response at both 5 and 10 Hz, while state 11 did not show any significant effect of variation in neuronal spiking frequency. A repeated-measures ANOVA carried out only on 3xTgAD mice further confirmed this result (N_3xTgAD-ChR2_ = 12 mice; n = 72 runs; each animal underwent 2 sessions of 3 runs, 1 run per stimulation frequency [5, 10, and 20 Hz]) (Figures S5E–S5H). These results indicate that despite optogenetic stimulation in 3xTgAD mice causing multiple dynamic states of activity that do not differ from those elicited in the WT group, frequency-specific responses to EC theta modulation are disrupted. These findings provide causal evidence of how EC frequency-dependent brain states can be disrupted during disease.

## Discussion

We provide sufficient causal evidence that in healthy WT mice, driving theta oscillations in EC neuronal spiking causes temporally overlapping but distinct dynamic brain states to emerge (Figures 2 and 3). Crucially, we demonstrate that these distinct dynamic states preferentially respond to inducing oscillations at different theta frequencies (either 5 or 10 Hz, but not 20 Hz) (Figure 4), revealing how multiple dynamic states can emerge from frequency modulation of the EC. We then show that these distinct dynamic states can rely on different anatomical circuits (Figures 4G and S4), suggesting a role for anatomical connections in the organization of dynamic brain states. Notably, we further show that in a transgenic model of EC network dysfunction (3xTgAD) (Mastrangelo and Bowers, 2008; Oddo et al., 2003; Yeh et al., 2011), the multiplicity of EC-driven dynamic states is intact, but there is a lack of frequency-dependent responses (Figure 5), thus demonstrating the necessity of an intact EC physiological function to underpin frequency-dependent dynamic state responses.

By bridging cell-specific neuronal modulation and brain-wide dynamics, the findings presented here provide key insights into the neuronal basis underlying dynamic brain states that are commonly observed in humans. Recent human neuroimaging studies have shown that the human brain organizes into frequency-tuned dynamic states, both at rest (Baker et al., 2014; Higgins et al., 2021; Vidaurre et al., 2017, 2018) and during tasks (Quinn et al., 2018), thus suggesting that the transient, frequency-dependent synchronization of neuronal activity may play a key role in shaping dynamic brain function and thus flexible cognition. However, previous photostimulation studies in rodents explicitly manipulating neuronal populations via frequency modulation have neglected transient brain events. In fact, these studies have shown that frequency modulation of a single neuronal population results in static magnitude changes in regional activation and functional connectivity (Chan et al., 2017; Leong et al., 2016; Liu et al., 2015). Here, by combining ofMRI in mice with a novel computational approach (HMMs), we bridge the gap between the human and the rodent literature. By decoding transient states of brain-wide activity elicited via single-area neuronal spiking, we demonstrate that local frequency modulation elicits brain-wide dynamic states that preferentially respond to different modulation frequencies. These results causally link activity in EC neuronal spiking with frequency-dependent dynamic states of brain function, providing key insights into one of the neuronal mechanisms that may allow flexible switching in brain activity to match the fast varying environmental demands (van der Meer et al., 2020).

The results of this work also provide initial support to the theory of brain communication via neuronal frequency-division multiplexing (Akam and Kullmann, 2014). Frequency-division multiplexing is a communication strategy that can allow selective broadcast of information to multiple targets through frequency variation in the source of activity. Different targets would then be able to read out different signals from the same activity pattern because of an underlying input frequency preference (or gain-response function). Here, we show that frequency modulation in the source of activity can elicit specific channels of brain communication (frequency-specific dynamic brain states), thus providing initial support to the theory of brain communication via frequency-division multiplexing.

While combining optogenetics with fMRI allows us to bridge cell-specific activity and brain-wide function, ofMRI also presents some limitations. Brain imaging techniques have an intrinsic trade-off between a second-resolved temporal resolution and brain-wide imaging. Therefore, while using HMM gives us access to fast transitions in BOLD fluctuations amplitude and coupling, it cannot overcome the limited temporal resolution of fMRI. Another limitation is that perturbing genetically targeted excitatory neurons inevitably also led to an immediate engagement of local perisynaptic activity in the form of excitatory and inhibitory loops (Logothetis, 2010). Hence, photostimulation of the entorhinal cortex excitatory circuits is likely to induce non-linear responses within the circuits that may locally further modulate the input frequencies. However, our approach also comes with advantages. Implementing HMMs (Vidaurre et al., 2017, 2018) allows us to disentangle dynamics of spatially and frequency distinct networks otherwise temporally overlapping and not observable using standard analyses or invasive techniques such as single-area electrophysiology. Furthermore, using HMMs, together with manipulations of the stimulation frequency, allows us to characterize between brain states preferentially responding at specific theta frequencies (states 10 and 11) and brain states with long-scale response amplification (state 6), suggesting that different underlying neural mechanisms are taking place. Our experimental and computational approaches allows us to establish the causal sufficiency (Adamantidis et al., 2015) of neuronal frequency modulation as a mechanism for generating multiple and specific brain-wide dynamic states. Future research should leverage optogenetics to establish the role of other neural populations (e.g., GABAergic interneurons) in driving, selecting, or silencing distinct brain dynamic states.

### Limitations of the study

From a methodological point of view, using principal-component analysis (PCA) as a preprocessing step before the application of the HMM is not optimal (Vidaurre, 2021). In particular, while the objective of the HMM is to find dynamic changes in covariance, PCA is built on the average covariance, therefore ignoring any non-stationarity in the data. As discussed in Vidaurre (2021) in detail, this can introduce certain biases and lack of sensitivity when non-stationarities occur within the discarded principal components (in this case, in the discarded 50% of the variance). In this work, however, we used PCA as a simpler alternative, since this approach has been used in other works successfully and it is easier to relate to previous work in fMRI (Stevner et al., 2019; Vidaurre et al., 2017). Note that HMM-PCA could not be applied here trivially in substitution of the present approach, since we were interested in modeling changes in signal amplitude as well as covariance, and HMM-PCA only models the covariance; see Figure S2E for a quantitative evaluation of how non-stationary PCA decompositions would relate to one another.

### Conclusions

To further our knowledge of how the brain supports flexible cognition, we need to ultimately understand causal cell-type-specific mechanisms supporting dynamic brain function. Combining optogenetics with concurrent fMRI allows us to causally manipulate cell-specific activity to understand large-scale dynamics in brain function. In this work, we considered the EC, a well-known hub in the brain memory system known to rely on theta rhythms to mediate memory encoding and recall in mice (Mizuseki et al., 2009) and known to support fine temporal memory retrieval in humans (Montchal et al., 2019). We used this structure and its anatomical projections in mice to causally test whether modulating excitatory neurons with distinct theta frequencies cause distinct dynamic brain states to emerge and to respond in a frequency-dependence fashion. By combining ofMRI with HMMs and by establishing causality between frequency variations in single-area activity and frequency-dependent dynamic states, this work provides cellular insight into a neuronal mechanism that may underpin frequency-dependent brain responses in flexible behavior.

## STAR★Methods

### Key resources table

**Table:** 

REAGENT or RESOURCE	SOURCE	IDENTIFIER
**Bacterial and virus strains**		

AAV5-CaMKIIa-hChR2(H134R)-mCherry	Vector Core at the University of North Carolina	N/A
AAV5-CaMKIIa-mCherry	Vector Core at the University of North Carolina	N/A

**Deposited data**		

Raw fMRI data in BIDS format	Openeuro.org	https://openneuro.org/datasets/ds002134

**Experimental models: organisms/strains**		

B6;129-Psen1^tm1Mpm^ Tg(APPSwe,tauP301L)1Lfa/Mmjax	The Jackson Laboratory	JAX: 34830

**Software and algorithms**		

FMRIB’s Software Library, FSL	Smith et al., 2004	https://fsl.fmrib.ox.ac.uk/fsl/fslwiki
HMM-MAR	Vidaurre et al., 2017	https://github.com/OHBA-analysis/HMM-MAR
PALM	Winkler et al., 2016	https://fsl.fmrib.ox.ac.uk/fsl/fslwiki/PALM
Original code	This paper	https://doi.org/10.5281/zenodo.5541144

### Resource availability

#### Lead contact

Further information and requests for resources and reagents should be directed to and will be fulfilled by the lead contact, Joanes Grandjean (Joanes.Grandjean@radboudumc.nl).

#### Materials availability

This study did not generate new unique reagents.

### Experimental model and subject details

#### Sample

All experiments performed in Singapore Bioimaging Consortium, A^∗^STAR, Singapore, were in accordance with the ethical standards of the Institutional Animal Care and Use Committee (A^∗^STAR Biological Resource Centre, Singapore, IACUC #171203). Male 3xTgAD (B6;129-Psen1tm1Mpm Tg(APPSwe,tauP301L)1Lfa/Mmjax) and WT mice on the same background strain (129sv/c57bl6) were used for the ofMRI experiments. The colonies of both 3xTgAD and WT mice were maintained ‘in-house’ through the pairing of homozygous individuals. Mice were housed in cages of up to five, with same-sex and genotype cage-mates in a pathogen-free environment, kept at a 45%–65% humidity, under a 12:12-hour light-dark cycle and room temperature, with *ad-libitum* access to food and water. The animals were split into three groups: wild-type transfected with mCherry control (N_WT-mCherry_ = 9), wild-type transfected with Channelrhodopsin 2 CaMKIIa-ChR2-mCherry (N_WT-ChR2_ = 10), and 3xTgAD transfected with Channelrhodopsin 2 (N_3xTgAD-ChR2_ = 12). The wild-type mCherry control group was imaged at 3 months. Both groups transfected with Channelrhodopsin 2 were imaged at 3 and 6 months (N_WT-ChR2_ = 8; N_3xTgAD-ChR2_ = 10, group attrition due to post-imaging recovery complications).

### Method details

#### Viral injection and fiber implant

Optogenetic surgery procedure is described in Mandino et al. (2020). Briefly, WT and 3xTgAD mice (∼30 g, ofMRI dataset: N = 31 mice (N_WT-mCherry_ = 9, N_WT-ChR2_ = 10, N_3xTgAD-ChR2_ = 12) were anaesthetised with a mixture of ketamine/xylazine (ketamine 75 mg/kg, xylazine 10 mg/kg). The head was shaved and cleaned with three wipes of Betadine® and ethanol (70%). Lidocaine was administered subcutaneously, *in situ*. To avoid hypothermia, animals were placed on a warm pad and the head fixated on the stereotaxic frame; protective ophthalmic gel was applied to the eyes to avoid dryness. After removing the scalp, a craniotomy was performed in the left hemisphere, with a hand-held drill (burr tip 0.9 mm^2^) ; coordinates from bregma and midline: −2.8 from bregma, +4.2 from the midline. AAV injection into the EC was carried out through this craniotomy, at a depth of −2.8 to −2.7 mm from the brain surface; the fiber-optic cannula positioning reached −2.6 mm from the surface. Coordinates were taken according to the Paxinos mouse brain atlas (Paxinos and Franklin, 2004). The injection of adeno-associated virus (AAV) was performed in the target location using a precision pump (KD Scientific Inc., Harvard Bioscience) with a 10 μL NanoFil syringe with a 33-gauge beveled needle (NF33BV-2). The AAV used (Deisseroth, 2011) (AAV5-CaMKIIa-hChR2(H134R)-mCherry (N_WT_ = 10, N_3xTgAD_ = 12), AAV5-CaMKIIa-mCherry (N_WT-mCherry_ = 9), titer 1-8x10^12^ vg/ml), were acquired from Vector Core at the University of North Carolina (USA). A total volume of 0.75 μL of the vector was injected in each mouse at a rate of 0.15 μl/min. To avoid backflow, the needle was kept in position for 10 minutes; after the needle extraction, a fiber-optic cannula (diameter 200 μm, 0.39 NA, length according to the injection site, diameter 1.25 mm ceramic ferrule) was lowered to the targeted region (Laser 21 Pte Ltd, Singapore; Hangzhou Newdoon Technology Co. Ltd, China). The cannula was fixed in place with dental cement (Meliodent rapid repair, Kulzer). Buprenorphine was administered post-surgically to each animal. Animal recovery took place on a warm pad.

#### Imaging: animal preparation

Animal preparation followed a previously established protocol (Grandjean et al., 2014). Anesthesia was induced with 4% isoflurane; subsequently, the animals were endotracheally intubated, placed on an MRI-compatible cradle and artificially ventilated (90 breaths/minute; Kent Scientific Corporation, Torrington, Connecticut, USA). A bolus with a mixture of Pancuronium Bromide (muscle relaxant, Sigma-Aldrich Pte Ltd, Singapore) and Medetomidine (Dormitor, Elanco, Greenfield, Indiana, USA) was provided subcutaneously (0.05 mg/kg). A maintenance infusion (0.1 mg/kg/hr) was administered 5 minutes later, with isoflurane reduced to 0.5%. Functional MRI was acquired 20 min following maintenance infusion onset to allow for the animal state to stabilize. Care was taken to maintain the temperature of the animals at 37°C using a feedback controlled water bath.

#### Optogenetics functional MRI

Functional imaging was performed on a 11.7T Biopsec system equipped with B-GA09S gradients, a 72 mm volume coil for excitation, and a 10 mm loop coil placed over the head for reception. The system was controlled using Paravision 6.0.1. The parameters for the rs-fmri data acquisition are as follows: the anatomical reference scan was acquired using FOV = 20 × 10 mm^2^, number of slices = 34, slice thickness = 0.35, slice gap = 0 mm, MD = 200 × 100, TR = 2000 ms, TE = 22.5 ms, RARE factor = 8, number of averages = 2. Functional scans were acquired using FOV = 17 × 9 mm^2^, FOV saturation slice masking non-brain regions, number of slices = 21, slice thickness = 0.45, slice gap = 0.05 mm, MD = 60 × 30, TR = 1000 ms, TE = 11.7 ms, flip angle = 50°, volumes = 720, bandwidth = 119047Hz. Field inhomogeneity was corrected using MAPSHIM protocol. Light stimulation was provided through a blue light laser (473 nm, LaserCentury, Shanghai Laser & Optic Century Co., Ltd; ∼12-15 mW/mm^2^ output with continuous light at the tip of the fiber, corresponding to ∼5 mW/mm^2^ inside the tissue) controlled by in-house software (LabVIEW, National Instruments). The fiber implant was connected via a low profile cord adaptor (Doric). Stimulation power was based on previous optimization in anesthetized mice (Grandjean et al., 2019). We did not find evidence of photostimulation-induced response in the mCherry control group at the photostimulation site. After an initial 50 s of rest as a baseline, 10 ms light pulses were applied at 5, 10 or 20Hz for 10 s followed by a 50 s rest period, in a 10-block design fashion. An additional 60 s of rest were recorded after the last block of stimulation. The photostimulation corresponds to a 20, 10, and 5% duty cycle, respectively. The experimental groups (3xTgAD and WT mice with ChR2-mCherry) and the negative control group (wild-type mice with mCherry alone) underwent the same imaging protocol, i.e., 5Hz, 10Hz and 20Hz evoked fMRI scans in pseudorandomized order, balanced between groups. The negative control group was imaged with the same imaging protocol as the experimental groups to exclude potential heating and/or vascular photoreactivity artifacts (Christie et al., 2013; Rungta et al., 2017), and to account for photostimulation of the visual system and potential tissue heating artifacts. This resulted in a ofMRI dataset with N = 31 mice (N_WT-mCherry_ = 9, N_WT-ChR2_ = 10, N_3xTgAD-ChR2_ = 12), for a total of n = 147 runs. Additionally, in order to exclude abnormal behavior induced by the photostimulation protocol such as seizures, all animals underwent the three stimulation sessions (5Hz, 10Hz, and 20Hz) again while awake and freely walking in a behavior-chamber for 10 min each. No animal displayed seizures or abnormal behavior in response to photostimulation.

#### fMRI preprocessing

Images were processed using a protocol optimized for the mouse and corrected for spikes (*3dDespike, AFNI*; Cox, 1996), motion (*FSL mcflirt*; Smith et al., 2004)*;* and B1 field inhomogeneity (*FSL fast*). Automatic brain masking was carried out on the EPI using *FSL bet*, following smoothing with a 0.3 mm^2^ kernel (*FSL susan*) and a 0.01Hz high-pass filter (*fslmaths*). Nuisance regression was performed using FIX. A study-specific classifier was generated for the ofMRI data based on 15 randomly-selected manually-classified runs (Zerbi et al., 2015). The EPIs were registered to the Allen Institute for Brain Science (AIBS) reference template ccfv3 using SyN diffeomorphic image registration (*antsIntroduction.sh, ANTS*; Avants et al., 2014)). The AIBS atlas was resampled to 90 regions-of-interest by merging leafs (e.g., cortical layers) by branches (e.g., cortical area). The nomenclature, and abbreviations for the brain regions are in accordance with https://atlas.brain-map.org/.

#### Whole-cell patch recording of brain slices

Mouse brains were rapidly removed after decapitation and placed in high sucrose ice-cold oxygenated artificial cerebrospinal fluid (ACSF) containing the following (in mM): 230 sucrose, 2.5 KCl, 10 MgSO_4_, 0.5 CaCl_2_, 26 NaHCO_3_, 11 glucose, 1 kynurenic acid, pH 7.3, 95% O_2_ and 5% CO_2_. Coronal brain slices were cut at a thickness of 250 mm using a vibratome (VT1200S; Leica Biosystems) and immediately transferred to an incubation chamber filled with ACSF containing the following (in mM): 119 NaCl, 2.5 KCl, 1.3 MgCl_2_, 2.5 CaCl_2_, 1.2 NaH_2_PO_4_, 26 NaHCO_3_, and 11 glucose, pH 7.3, equilibrated with 95% O_2_ and 5% CO_2_. Slices were allowed to recover at 32°C for 30 minutes and then maintained at room temperature. Experiments were performed at room temperature. Whole-cell patch-clamp recordings were performed on EC pyramidal cells expressing ChR2-mCherry and were visualized using a CCD camera and monitor. Pipettes used for recording were pulled from thin-walled borosilicate glass capillary tubes (length 75 mm, outer Ø 1.5 mm, inner Ø 1.1 mm, WPI) using a DMZ Ziets-Puller (Zeitz). Patch pipettes (2–4 MW) were filled with internal solution containing (in mM): 105 K-gluconate, 30 KCl, 4 MgCl_2_, 10 HEPES, 0.3 EGTA, 4 Na-ATP, 0.3 Na-GTP, and 10 Na_2_-phosphocreatine (pH 7.3 with KOH; 295 mOsm), for both voltage- and current-clamp recordings. Photostimulation (460 nm) was delivered by an LED illumination system (pE-4000). Several trains of a square pulse of 20 ms duration with 5, 10, and 20Hz, were delivered respectively under current-clamp mode (I = 0) to examine whether the neurons were able to follow high-frequency photostimulation. After different frequencies of photostimulation were completed, neurons were shifted to voltage-clamp mode (at −60 mV), and a prolonged square pulse of 500 ms duration was delivered, to further confirm whether ChR2-induced current could be seen in the recorded neurons. The access resistance, membrane resistance, and membrane capacitance were consistently monitored during the experiment to ensure the stability and the health of the cell.

### Quantification and statistical analysis

#### Hidden Markov modeling of whole-brain ofMRI

BOLD ofMRI time-series were extracted from the AIBS atlas resampled to 90 regions-of-interest (https://atlas.brain-map.org/), and then reduced to a space of principal components accounting for 50% of the variance. In this reduced space, BOLD fMRI time-series were modeled using hidden Markov Models (HMMs; https://github.com/OHBA-analysis/HMM-MAR) as a sequence of visits to a finite set of transient states, each represented by a characterized pattern of activity and functional connectivity. Specifically, the HMM states were modeled as Gaussian distributions, described by mean and covariance — which relate, respectively, to the average pattern of BOLD activity when each state is active and its functional connectivity (Vidaurre et al., 2017).

We applied HMMs on temporally-concatenated subjects across experimental groups: healthy control group (N_WT-mCherry_ = 9), healthy active group (N_WT-ChR2_ = 10), and AD-like pathology active group (N_3xTgAD-ChR2_ = 12), thus a total of N = 31 mice, n = 153 runs (720 time-points for each ofMRI run; total of 110,160 time-points). In order to identify an adequate number of states, we fit the HMMs with different states number (10, 12, 14, 16, and 18) and, for each number, we repeated the fitting 3 times, thus resulting in 3 HMMs per state number. We then identified the best HMM configuration for this ofMRI dataset as the number of states that provided the highest similarity between repetitions with lowest average free energy (for each state number, this was calculated as the ratio between model similarity and average free energy across repetitions). This allowed us to identify a remarkably robust temporal organization for 14 states (see Figure S1A). Subsequent analyses were carried out on HMM with 14 states,on the repetition with the lowest free energy value. We also show that HMMs with different states number (12 and 16) were also capable of capturing brain dynamics induced by optogenetics (see Figures S2A and S2B).

Although the spatial parameters of the HMM states were inferred at the group level, each animal has its own characteristic state time course representing the subject-specific probability of each HMM state being active at each instant (here, an fMRI volume). In other words, the temporal parameters of the HMM across the 720 time-points of an ofMRI run are inferred at the subject-level. Statistical testing for inferences across groups or across stimulation frequencies, were carried out on these states’ time courses. Details are provided below.

#### Inference testing on HMM probabilities

All inference testing was carried out using Permutation Analysis of Linear Models (PALM: https://fsl.fmrib.ox.ac.uk/fsl/fslwiki/PALM; Winkler et al., 2016). The null distribution was characterized with 1,000 permutations. Statistical significance was established based on family wise error (FWE)-corrected p value.

When testing inferences on HMM responses to optogenetic stimulation (i.e., group comparisons: WT-ChR2 versus WT-mCherry), one-dimensional threshold-free cluster enhancement (TFCE) was applied, thus FWE-corrected p values were fully corrected across time. Also, in exploratory analyses, statistical significance was further corrected across *all* states tested, thus p values are FWE-corrected across time *and* states. All statistical significance results are plotted as -log_10_ of FWE-corrected p values. All statistical analyses were carried out in MATLAB 2018.

#### HMM response time-locked to optogenetics

Each ofMRI run comprised 10 stimulation blocks. In order to create HMM responses time-locked to stimulation, we considered, for each run, the HMM activation probability 10 s from beginning of stimulation, plus 10 s after the end of stimulation, for a total of 20 s (10 on, 10 off). We then calculated the median across the 10 blocks - as this is less affected by outliers and skewed data and is usually the preferred measure when the distribution is not symmetrical. Short-scale HMM responses were studied by contrasting groups: WT-mCherry versus WT-ChR2, or WT-ChR2 versus 3xTgAD. Significance was assessed through permutation testing as explained above in “*Inference testing on HMM time-series*.” The results from this analysis can be found in Figures 3C, 5C, S2A, and S2B.

#### Long-scale variation in HMM responses

HMMs characterize time-series for each state across the whole ofMRI run: we can therefore assess how short-scale HMM responses vary across long-scales. In order to do this in an unbiased fashion, we used permutation-based statistical significance as a criteria to deem a HMM response to a single block significant or not. Long-scale HMM responses were studied by contrasting WT-mCherry group versus WT-ChR2 group. The results from this analysis can be found in Figure S3.

#### Frequency effect on time-locked HMM responses

Each subject underwent multiple ofMRI runs at different frequencies (5, 10, and 20Hz). In order to study the effect of frequency on time-locked HMM responses, a group-specific repeated-measure ANOVA was carried out (as implemented in FSL randomize: https://fsl.fmrib.ox.ac.uk/fsl/fslwiki/Randomise). Given the key role of theta rhythms in the EC-hippocampal circuit, our hypotheses predicted specific responses to each theta rhythm (5 and 10Hz). We therefore specifically contrasted 5Hz to 10 and 20Hz, and 10Hz to 5 and 20Hz, and carried out an overall F-test for the effect of stimulation frequency as well as post hoc t tests. Each set of three runs with frequencies at 5, 10, and 20Hz (also referred in the manuscript as *session*), was considered as an independent block, and block-aware permutation testing was carried out (combination of within-block and whole-block) (Winkler et al., 2015). Frequency effects on short-scale HMM responses were studied separately for WT-ChR2 group and for 3xTgAD group. The results from this analysis can be found for WT-ChR2 group in Figures 4A (F-test), 4C and 4D (t tests), and also in S2C and S2D; while for the 3xTgAD group can be found in Figures 5F and 5G (t tests).

#### Group by frequency effect on HMM responses

In order to study group by frequency interactions (theta rhythms) on time-locked HMM responses, a mixed-design ANOVA was carried out using FSL PALM (Winkler et al., 2016). Raw data can be seen in Figures S5A and S5B. Group (WT versus 3xTgAD) was a between-subjects factor (fixed), while frequency (5Hz to 10 and 20Hz, and 10Hz to 5 and 20Hz) was a within-subject factor (random). An overall F-test of the interaction effect with both stimulation contrasts was carried out as well as post hoc t tests (for 5Hz to 10 and 20Hz: WT > 3xTgAD and 3xTgAD > WT; and, for 10Hz to 5 and 20Hz: WT > 3xTgAD and 3xTgAD > WT). Each set of three runs with frequencies at 5, 10, and 20Hz (also referred in the manuscript as *session*), was considered as an independent block, and block-aware permutation testing was carried out (combination of within-block and whole-block) (Winkler et al., 2015). The results from this analysis can be found in Figure S5C (F-test) and in Figure S5D (t tests).

#### General linear modeling of whole-brain ofMRI

Brain hemodynamic fluctuations during ofMRI were also examined using a traditional general linear model (GLM) framework (*FSL fsl_glm*). The stimulation paradigm and its first derivative were convolved using the default gamma function and used as regressors in the analysis, with motion parameters as covariates. Threshold-free cluster enhanced (TFCE) FWE-corrected p values were deemed significant when below 0.05 after permutation testing. This analysis was carried out on the WT-ChR2 group only. The results from this analysis can be found in Figure S2E.

#### Association with EC monosynaptic projection

The pattern of EC monosynaptic tracing projection was derived from the publicly available data of the Allen Brain Institute (Oh et al., 2014). Magnitude values were normalized via rank-based inverse normal transformation. Statistical association between EC projection pattern and spatial activity pattern of HMM states of interest was carried out using PALM (Winkler et al., 2016). The null distribution was characterized with 1,000 permutations. Statistical significance was established based on FWE-corrected p value. The results from this analysis can be found in Figures 3A, 3D, and Figure 5E.

#### Structural-to-functional mapping

In order to characterize the anatomical directed circuits that may underlie different brain activity patterns of frequency-dependent HMM states, we used the anatomical projection matrix of directed monosynaptic connection (derived from the publicly available data of the Allen Brain Institute (Oh et al., 2014)) to predict HMM states amplitude patterns. Predictor variables, with columns being different brain regions and, for each column, each row value being the magnitude of tracing projection to a target region, were normalized via rank-based inverse normal transformation and used to predict HMM state amplitude levels across brain regions (response variable). We did this using leave-one-out cross-validation regression with Ridge regularisation (as implemented in the FSL package FSLNets: https://fsl.fmrib.ox.ac.uk/fsl/fslwiki/FSLNets), and calculated out-of-sample explained variance (observed versus predicted). The lambda value (Lagrange multiplier) was estimated through an inner loop. Statistical significance of the out-of-sample prediction was assessed with 1,000 permutations. The estimated regression betas were then used to display the underlying estimated circuit of directed anatomical connections. Specifically, estimated regression coefficients were extracted and averaged across folds. These values were then dot-multiplied by the whole-brain directed connectivity matrix in order to characterize a directed connectivity matrix underlying a specific state of activity. To aid interpretation, only positive connections were considered and visualized as a circle graph. The results from this analysis can be found in Figures 4G and S4.

## Data Availability

• Raw MRI data have been deposited at OpenNeuro and are publicly available as of the date of publication. Accession numbers are listed in the Key resources table. All other data reported in this paper will be shared by the lead contact upon request. • All original code has been deposited at Zenodo and is publicly available as of the date of publication. DOIs are listed in the Key resources table. • Any additional information required to reanalyze the data reported in this paper is available from the lead contact upon request.

## Supplemental information

Document S1. Figures S1–S5

Document S2. Article plus supplemental information
