# Supplementary material for: Frequency modulation of entorhinal cortex neuronal activity drives distinct frequency-dependent states of brain-wide dynamics
Source: Cell Rep. 2021 Nov 2;37(5):109954. doi: 10.1016/j.celrep.2021.109954 (PMC8609366; doi:10.1016/j.celrep.2021.109954)
Supplement: Document S1. Figures S1–S5 [file mmc1.pdf]

Cell Reports, Volume *37*

Supplemental information

Frequency modulation of entorhinal cortex neuronal activity drives distinct frequency-dependent states of brain-wide dynamics

Piergiorgio Salvan, Alberto Lazari, Diego Vidaurre, Francesca Mandino, Heidi Johansen-Berg, and Joanes Grandjean

Figure S1

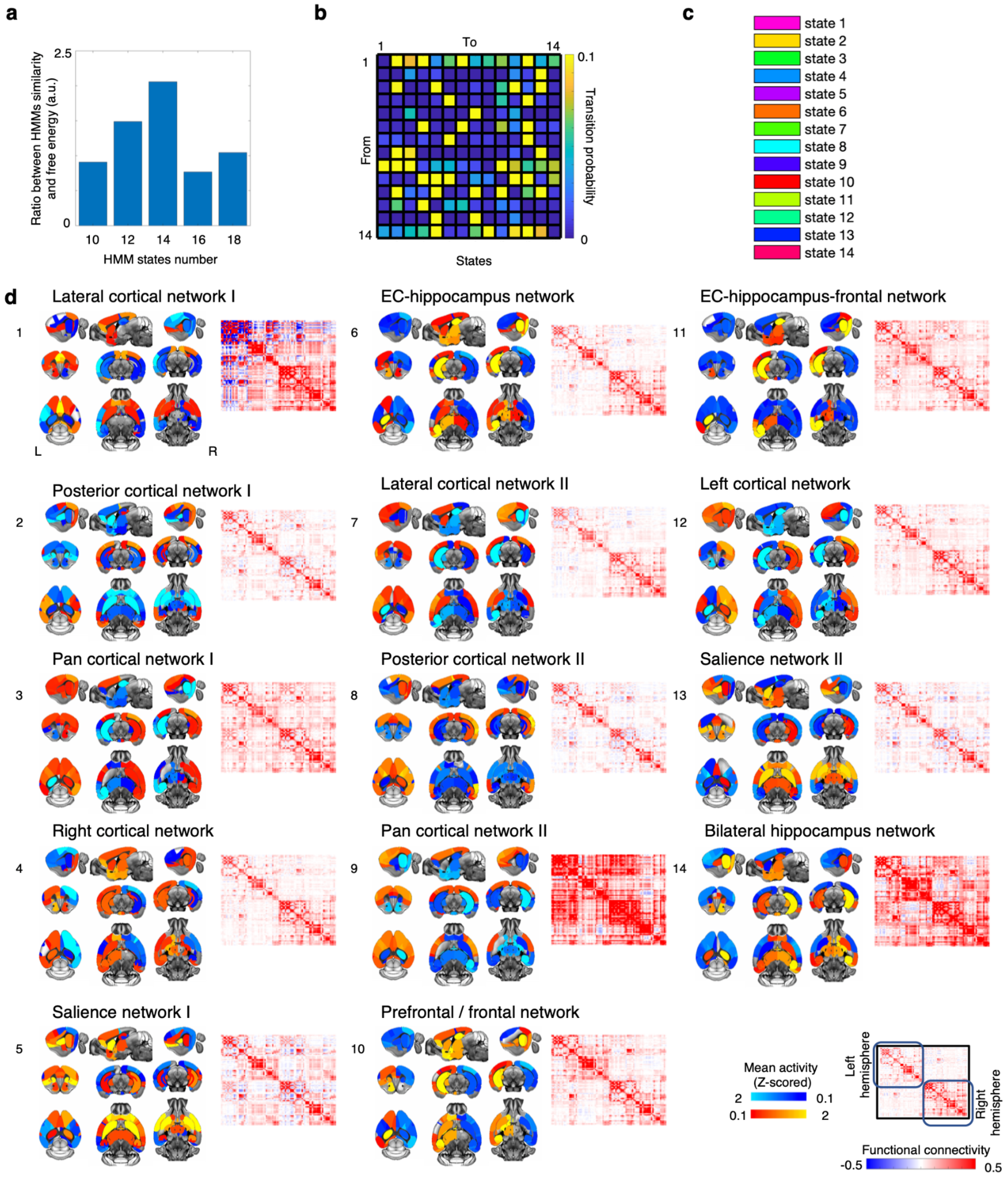

Figure S1 Brain states of whole-brain hemodynamic activity in response to optogenetic stimulation. Related to Fig.2. Hidden Markov models (HMMs) were used in order to characterize fast variations in whole-brain BOLD fMRI fluctuations. Multiple HMM were fitted with different states numbers (10, 12, 14, 16, and 18 states) across all subjects and runs. Each separate HMM was fit on N = 31 mice, n = 153 runs ($N_{WT\text{-}mCherry}$ = 9 mice, n = 27 runs acquired in 1 session; $N_{WT\text{-}ChR2}$ = 10 mice; n = 54 runs acquired into 2 sessions; $N_{3xTgAD\text{-}ChR2}$ = 12 mice; n = 72 runs acquired in two session; total: N = 31 mice, n = 153 runs). Each HMM state number configuration was assessed 3 times in order to calculate model similarity. The best HMM state number was identified based on the ratio between model similarity and average free energy. Results are shown in **a**): higher similarity as well as lower free energy would push the bar

higher. A state number of 14 led to models with greater similarity and lower free energy. For HMM with 14 states and lowest free energy. **b**) Showing matrix of transition probability between the 14 states. (The diagonal is displayed as zeros in order to ease interpretation of between states transitions). **c**) Color legend for matching HMM states parameters from panel **d**) with **Fig. 2-5** in the main text. **d**) For each HMM state, showing proposed state name (Gozzi and Schwarz, 2016), mean activity map (in 2D slices; for each state values are normalised across brain regions) and functional connectivity matrix.

Figure S2

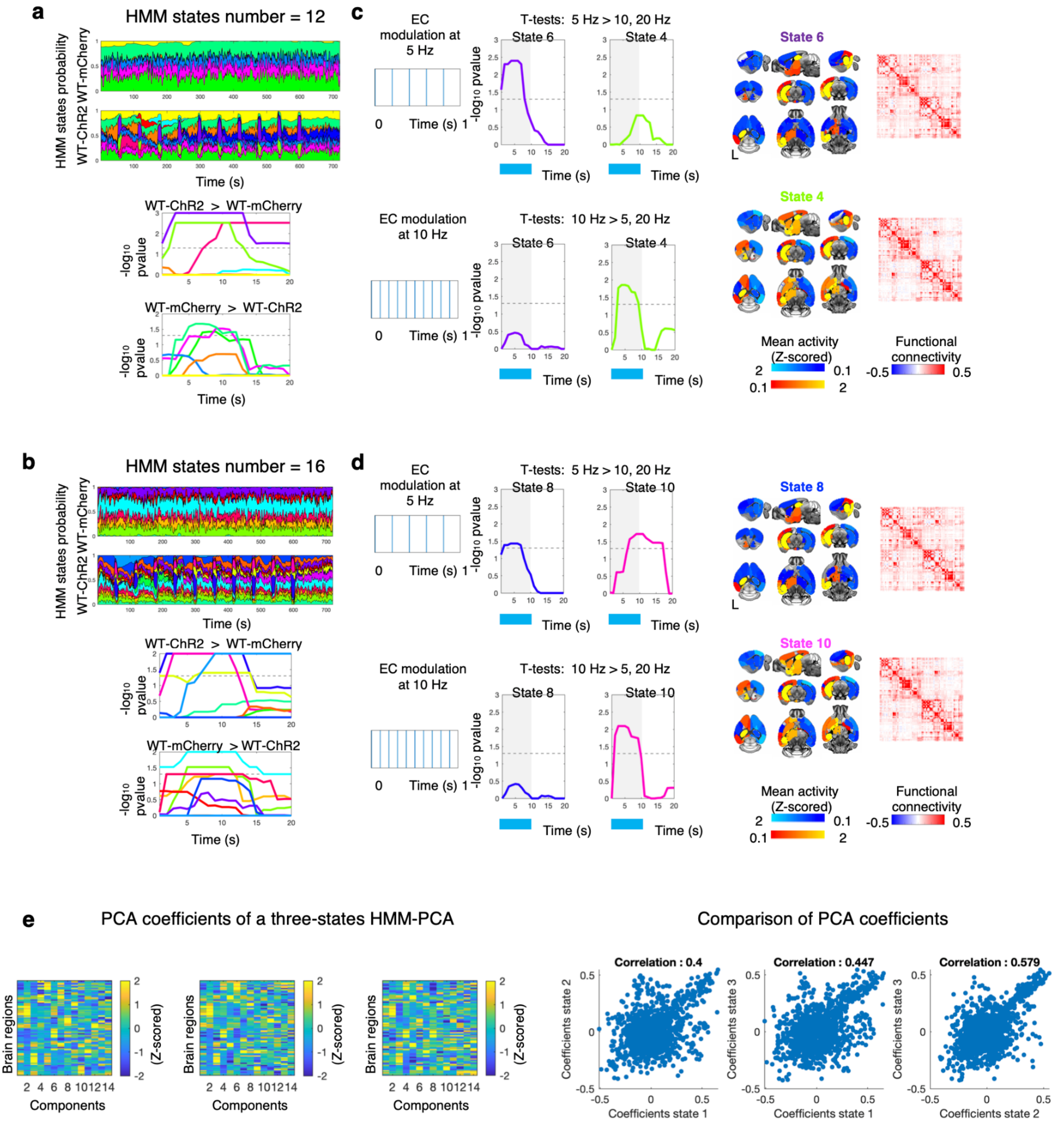

Figure S2 HMMs with different states number and General Linear Modelling. Related to Fig.2. **a**), **b**), **c**), and **d**) show reproducibility of results across HMMs with different states number: for **a**) and **c**), states number = 12, while for **b**) and **d**), states number = 16. **a**) and **b**) show group-average HMM states probability for both wild-type mCherry group and wild-type ChR2 group, as well as statistical results from group comparisons. (This figure relates to the main analysis displayed in **Fig. 3c**). In **c**) and **d**) showing results from repeated measures ANOVA post-hoc T-tests (as -$\log_{10}$ FWE-corrected p-values) of the two contrasts 5 Hz > 10, 20 Hz), and 10 Hz > 5, 20 Hz). (This figure relates to the main analysis displayed in **Fig. 4d, f**). Brain maps show HMM states of interest (and relate to the main maps displayed in **Fig. 3d**). WT = wild-type. **e**) Application of the HMM-PCA approach to quantify the extent of which stationary

PCA would differ from a local, state-based PCA decomposition. In the limit case, if all the state-based PCA decompositions were exactly equal, it would be equivalent to doing a single stationary PCA. Here, in the HMM-PCA run, 14 components were selected to mirror the number of components used in the HMM runs throughout the paper. Left: Showing the PCA coefficients of a three-state HMM-PCA run. Right: scatter plots (and correlation coefficients) comparing the PCA coefficients for each pair of states. As observed, the correlations are high, meaning that the PCA decompositions (when done in a non-stationary way) are not that different between states. This suggests that a stationary PCA reduction, however suboptimal, is still reasonable.

Figure S3

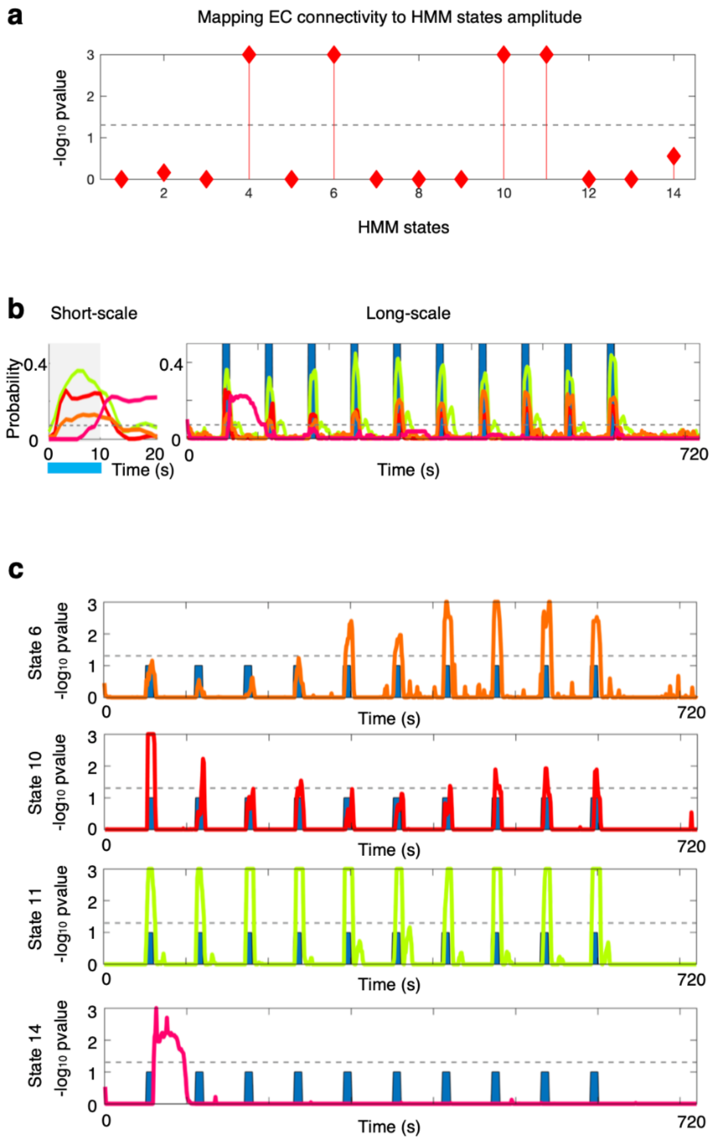

Figure S3 Optogenetic stimulation of EC causes short-scale and long-scale changes in brain network dynamics. Related to Fig.3. HMM allows to characterize fast-scale (or time-locked) as well as long-scale responses (or slow variations across fMRI run) in whole-brain hemodynamic activity. Results from analyzing ChR2 and control groups ($N_{WT\text{-}ChR2}$ = 10 mice; n = 54 runs; each animal underwent 6 runs acquired in 2 sessions; mCherry; $N_{WT\text{-}mCherry}$ = 9 mice, n = 27 runs; each animal underwent 3 runs acquired in 1 session; all stimulation frequencies combined). **a**) Showing -log10 FWE-corrected p-values of regressing EC outgoing monosynaptic connectivity pattern against all 14 HMM states' activity patterns. Significance values are FWE-corrected across all 14 states tested. Dotted line indicates the FWE-corrected statistical threshold. **b**) For WT-ChR2 group only, showing group-average HMM states activation probability time-locked to stimulation (left) and variations across stimulation blocks (right). Dotted line indicates the average activation level across all HMM states. **c**) Showing -log10 FWE-corrected p-values of group difference (WT-ChR2 > WT-mCherry) in HMM states activation probability across time. Dotted line indicates the statistical threshold.

Figure S4

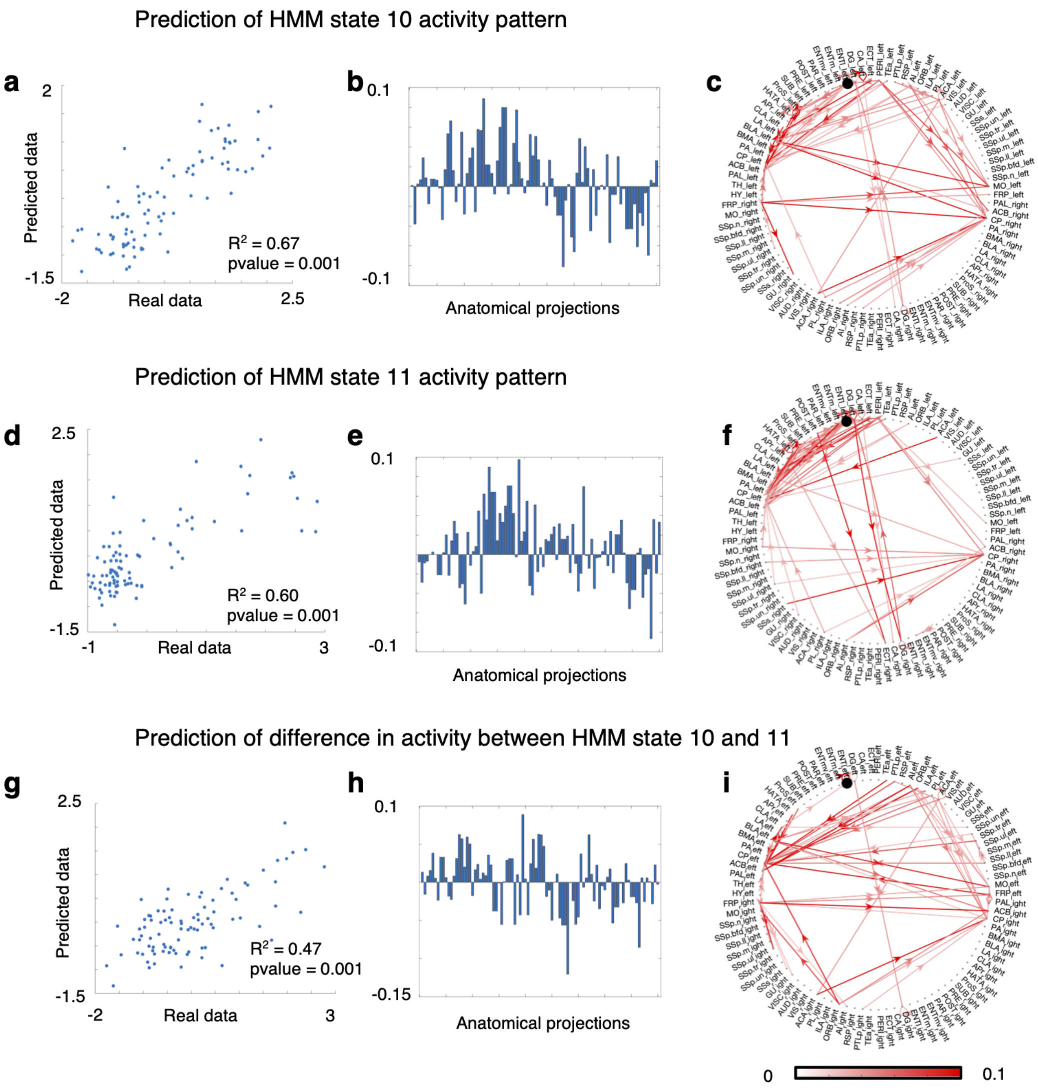

Figure S4 Different anatomical circuits predict activity patterns in distinct HMM states. Related to Fig.4. Cross-validated Ridge regression was used together with publicly available tracing data (see Methods) to predict HMM mean activity map of state 10. **a**) Scatter plot of real vs predicted values for *state 10* mean activity values. Average regression coefficients across folds were then extracted **b**) and plotted its positive values as a directed network (circular graph) **c**). The same approach was carried out for HMM state 11. Panels **d**), **e**), **f**), respectively mirror panels **a**), **b**), **c**) for *state 11*. Panels **g**), **h**), **i**), respectively mirror panels **a**), **b**), **c**) for the *difference between state 10 and 11*. Colorbar indicates the strength of the anatomical connections for panels **c**), **f**), **i**).

Figure S5

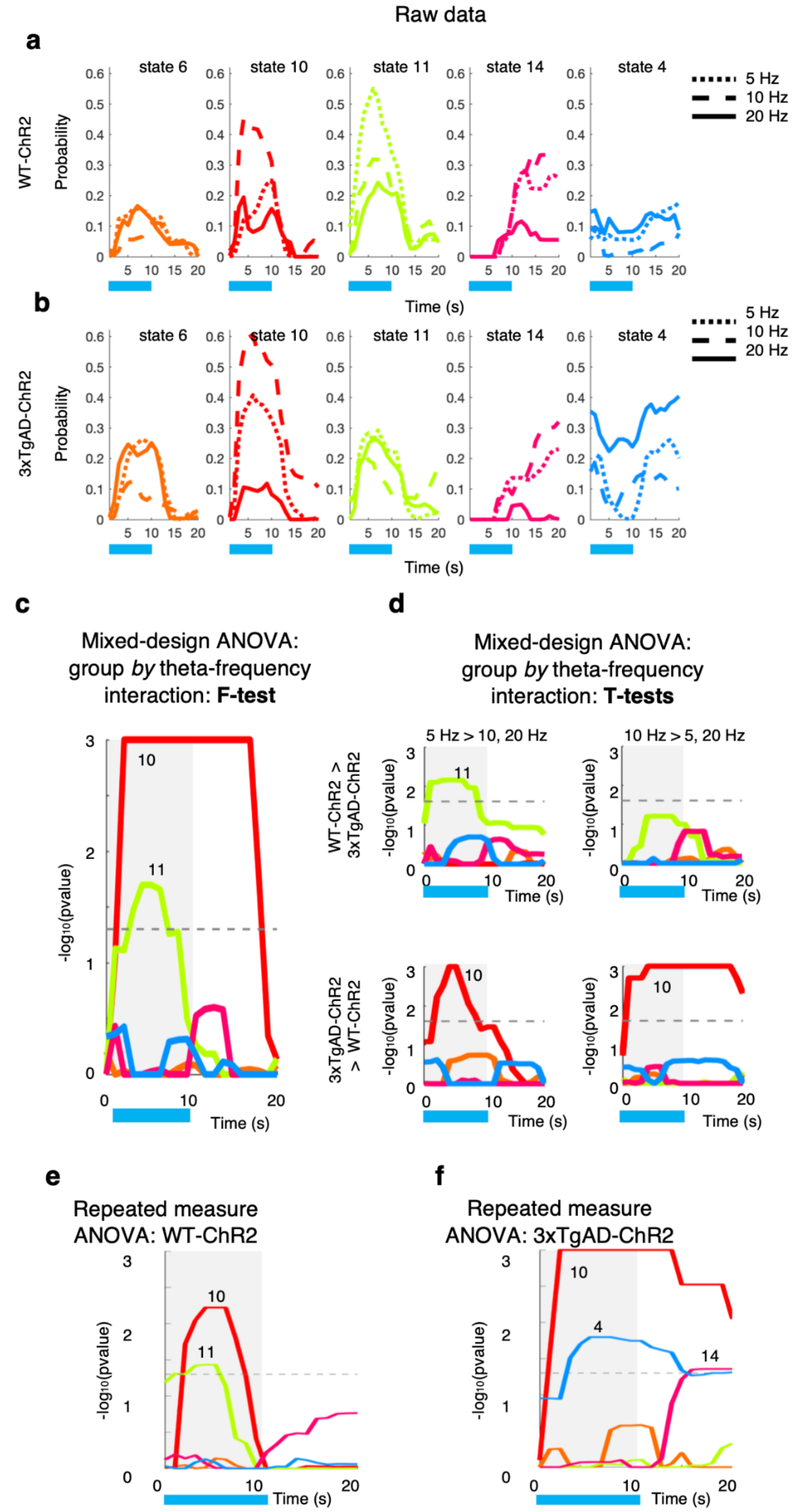

Figure S5 Groups by theta-frequencies interaction in HMM activation probability. Related to Fig.5. Showing results of group by beta-frequency interaction between the WT-ChR2 group and the 3xTgAD-ChR2 group (WT-ChR2 group and 3xTgAD-ChR2 group ($N_{WT\text{-}ChR2}$ = 10 mice; n = 54 run; $N_{3xTgAD\text{-}ChR2}$ = 12 mice; n = 72 runs; for both groups, each animal underwent 2 sessions of 3 runs, one run per stimulation frequency (5, 10, 20Hz)). **a**), **b**) Showing group-average HMM activation probability time-locked to optogenetic stimulation and divided by *group* (**a**) WT-ChR2 group, **b**) 3xTgAD-ChR2 group) and *frequency* (dotted line: 5Hz; dashed line: 10Hz; solid line: 20Hz). 3xTgAD-ChR2 group and WT-ChR2 group were compared via a mixed-design ANOVA testing the interaction of

groups *by* theta-frequencies. Statistical analyses were carried out on WT-ChR2 group and 3xTgAD-ChR2 group ($N_{WT\text{-}ChR2}$ = 10 mice; n = 54 run; $N_{3xTgAD\text{-}ChR2}$ = 12 mice; n = 72 runs; for both groups, each animal underwent 2 sessions of 3 runs, one run per stimulation frequency (5, 10, 20Hz)). **f**) Showing -$\log_{10}$ FWE-corrected p-values for an overall interaction effect of group *by* theta-frequencies on HMM states activation probability (F-test results). **d**) Showing -$\log_{10}$ FWE-corrected p-values for post-hoc T-tests of interaction analysis. Dotted line in **c** and **d** indicates the statistical threshold. To confirm the results of the interaction analysis shown in **Fig. 4f, g**, two separate repeated measure ANOVA were carried out independently in the WT-ChR2 group ($N_{WT\text{-}ChR2}$ = 10 mice; n = 54 run) and in the 3xTgAD-ChR2 group ($N_{3xTgAD\text{-}ChR2}$ = 12 mice; n = 72 runs). **c**) Showing -$\log_{10}$ FWE-corrected p-values of F-test for effect of theta-frequencies on in the WT-ChR2 group; results from post-hoc T-tests are shown in **e**). Plot c) is also shown in Fig. 4a. Here is reported again for direct comparison with the 3xTgAD-ChR2 group. **d**) Showing -$\log_{10}$ FWE-corrected p-values of F-test for effect of theta-frequencies on in the 3xTgAD-ChR2 group; results from post-hoc T-tests are shown in **f**). As a further post-hoc test, we also tested whether within each single-group there was a significant effect of theta-frequencies. **c**) Showing -$\log_{10}$ FWE-corrected p-values of F-test for effect of theta-frequencies only in the WT-ChR2 group (results from post-hoc T-tests are shown in **Fig. 4d, f**, and in **Fig. 5f**). Panel f) is also shown in **Fig. 4a**. **d**) Showing -$\log_{10}$ FWE-corrected p-values of F-test for effect of theta-frequencies only in the 3xTgAD-ChR2 group; results from post-hoc T-tests are shown in **Fig. 5g**). Dotted line in **c-f** indicates the statistical threshold.
